# Accident severity prediction modeling for road safety using random forest algorithm: an analysis of Indian highways

**DOI:** 10.12688/f1000research.133594.1

**Published:** 2023-05-15

**Authors:** Humera Khanum, Anshul Garg, Mir Iqbal Faheem

**Affiliations:** 1 School of Civil Engineering, Lovely Professional University, Phagwara, Punjab, 1444411, India; 2 Civil Engineering Department, Symbiosis Institute of Technology, Pune Campus, Symbiosis International (Deemed University), Pune, Maharashtra, 412115, India; 3 Civil Engineering Department, Deccan College of Engineering and Technology, Hyderabad, Telangana, 500001, India

**Keywords:** Traffic Accidents, Accident Severity, Road Safety, Accident Prediction Modeling, Random Forest

## Abstract

**Background:** Road accidents claim around 1.35 million lives annually, with countries like India facing a significant impact. In 2019, India reported 449,002 road accidents, causing 151,113 deaths and 451,361 injuries. Accident severity modeling helps understand contributing factors and develop preventive strategies. AI models, such as random forest, offer adaptability and higher predictive accuracy compared to traditional statistical models. This study aims to develop a predictive model for traffic accident severity on Indian highways using the random forest algorithm.

**Methods:** A multi-step methodology was employed, involving data collection and preparation, feature selection, training a random forest model, tuning parameters, and evaluating the model using accuracy and F1 score. Data sources included MoRTH and NHAI.

**Results:** The classification model had hyperparameters ‘max depth’:  10, ‘max features’: ‘sqrt’, and ‘n estimators’: 100. The model achieved an overall accuracy of 67% and a weighted average F1-score of 0.64 on the training set, with a macro average F1-score of 0.53. Using grid search, a random forest Classifier was fitted with optimal parameters, resulting in 41.47% accuracy on test data.

**Conclusions:** The random forest classifier model predicted traffic accident severity with 67% accuracy on the training set and 41.47% on the test set, suggesting possible bias or imbalance in the dataset. No clear patterns were found between the day of the week and accident occurrence or severity. Performance can be improved by addressing dataset imbalance and refining model hyperparameters. The model often underestimated accident severity, highlighting the influence of external factors. Adopting a sophisticated data recording system in line with MoRTH and IRC guidelines and integrating machine learning techniques can enhance road safety modeling, decision-making, and accident prevention efforts.

## Introduction

Road accidents are a significant public health concern worldwide, with an estimated 1.35 million deaths caused by road traffic accidents each year. ^1^ Developing countries, such as India, are disproportionately affected, with over 150,000 fatalities reported annually. ^1^ Road safety is a major concern in India, with a large number of accidents and fatalities reported each year. According to the Ministry of Road Transport and Highways, there were 449,002 road accidents in India in 2019, resulting in 151,113 deaths and 451,361 injuries. ^2^ To understand the factors that contribute to accidents and to develop strategies to prevent them, accident severity modelling is a statistical technique used in the field of road safety.

The modelling process involves analyzing data on past accidents and identifying the factors that contributed to their occurrence and severity. These factors can include road conditions, weather, driver behaviour, and vehicle type, among others. The goal of accident severity modelling is to identify the factors that are most important in contributing to accidents and to develop evidence-based strategies to improve road safety and reduce accidents’ number and severity. ^3^^–^^6^

Statistical models, such as logit models and probit models, ^7^ have been widely used for predicting traffic accidents’ severity since the early 1990s. However, if assumptions imposed on these models are violated, results may be inaccurate. Artificial intelligence models, on the other hand, do not make any assumptions and are more adaptable. They are capable of handling intricate nonlinear relationships and generally offer higher predictive accuracy than statistical approaches. random forest (RF) is a powerful and versatile algorithm for accident severity prediction, with several advantages over other machine learning algorithms. It has been successfully applied in various contexts, and its performance can be further improved by tuning the key parameters and carefully pre-processing the data. ^8^^–^^11^

The performance of the random forest algorithm is significantly influenced by the selection of hyperparameters. ^12^

To optimize its performance, identifying the optimal parameter values is crucial. Previous research has predominantly utilized grid search to explore values within the parameter space. However, alternative approaches may be necessary to overcome the computational challenge of grid search in high-dimensional parameter spaces. ^13^ Random search and Bayesian optimization are effective alternatives to grid search for hyperparameter optimization in random forest, particularly in high-dimensional parameter spaces. ^13^

Accurate prediction of traffic accident severity is essential for improving emergency response, reducing fatalities, and minimizing injuries. To achieve this goal, accurate data, appropriate machine learning algorithms, and regular updates to the predictive models are necessary. Given the advantages of random forest models in terms of prediction accuracy and interpretability, they can be used as the primary predictive model for traffic accident severity on Indian highways. Several factors contribute to accidents on Indian highways, including poorly designed or maintained roads, speeding, and roadside hazards. Identifying these factors through accident severity modelling can help develop evidence-based strategies to improve road safety and reduce accidents’ number and severity. ^14^^–^^16^

Indian highways have a high incidence of accidents, and several contributing factors have been identified. These include road design and geometry, speed, roadside hazards, and driver behavior. Poorly designed or maintained roads, such as those with narrow or winding stretches, lack of markings, and poor road surfaces, increase the likelihood of accidents. ^17^ Speeding is a major factor in many accidents on Indian highways, which may result from a lack of enforcement, cultural norms, and driver attitudes. ^18^ Roadside hazards, such as trees, poles, and animals, are prevalent on Indian highways and increase the risk of collision. ^19^ Driver behavior, including drunk, distracted, and reckless driving, is also a significant contributor to accidents on Indian highways. ^15^ Addressing these factors is crucial to improving road safety and reducing accidents on Indian highways.

The main objective of our study is to develop a predictive model for the severity of traffic accidents on Indian highways. To achieve this goal, we have chosen random forest models due to their ability to provide accurate predictions and interpretability.

The findings of our study will be used to develop a predictive model for accident severity that can inform road safety policies and interventions. This model can be used to identify high-risk areas and to prioritize resources for accident prevention and mitigation.

### Study Areas

The study areas selected are the National Highways two stretches as mentioned below

**Figure 1.**
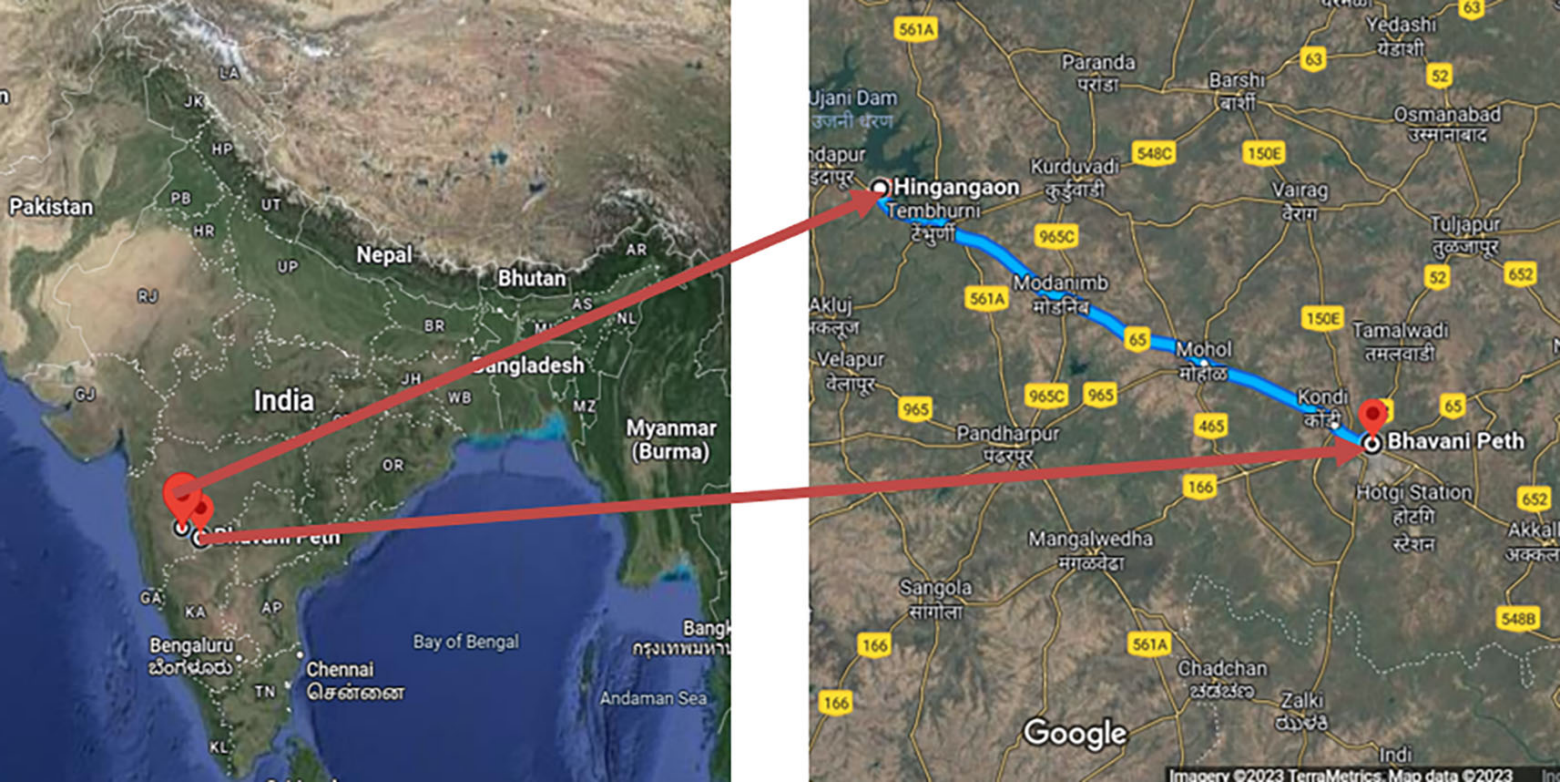
Pune-Sholapur Section of NH-9 in km.144/400 to Km. 249/000 in the State of Maharashtra.

**Figure 2.**
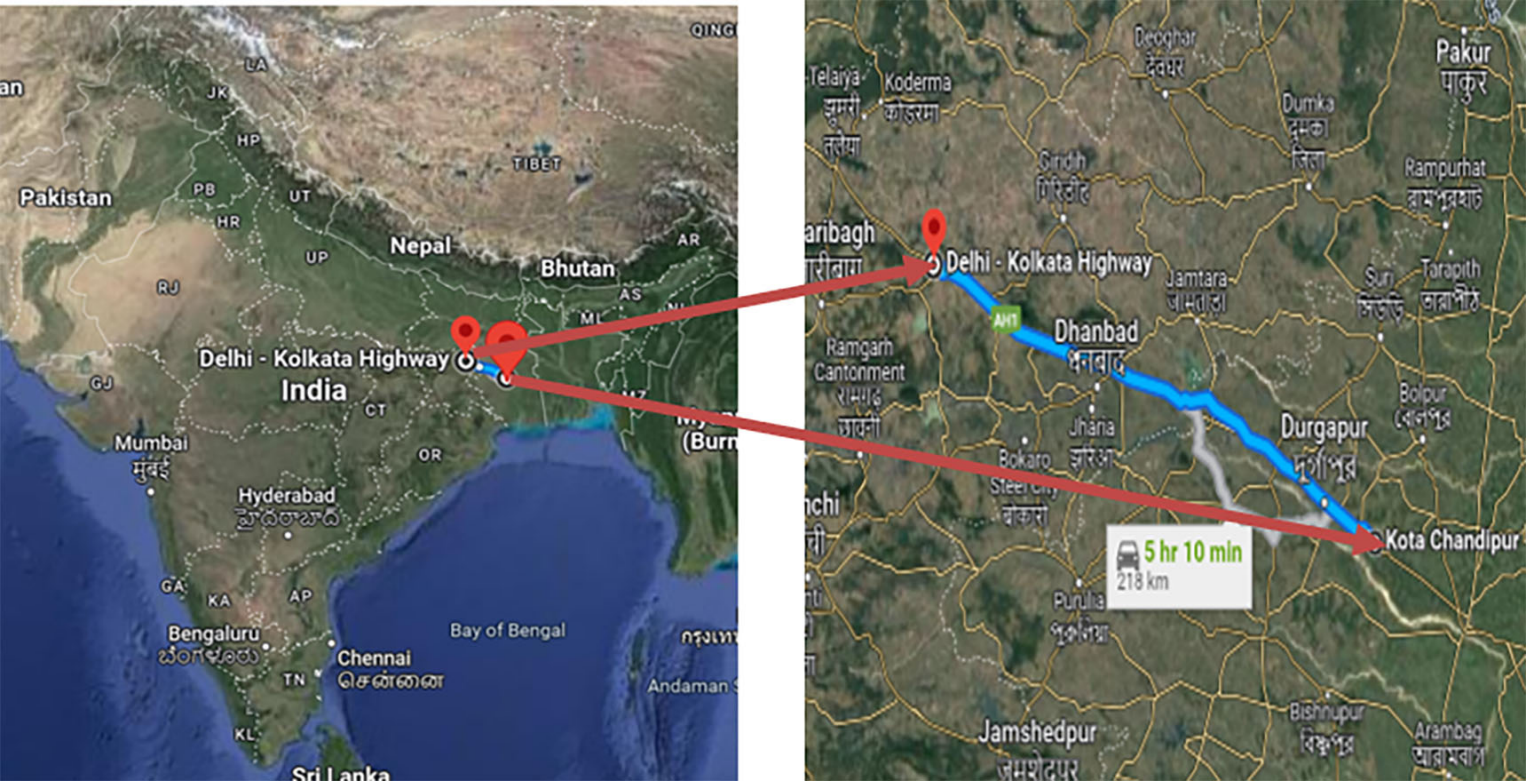
Six-Laning of Barwa-Adda-Panagarh Section of NH-2 from km 398.240 to km 521.120 including Panagarh Bypass in the States of Jharkhand and West Bengal.

1. Pune-Sholapur Section of NH-9 in km.144/400 to Km. 249/000 in the State of Maharashtra ( Figure 1).
2. Six-Laning of Barwa-Adda-Panagarh Section of NH-2 from km 398.240 to km 521.120 including Panagarh Bypass in the States of Jharkhand and West Bengal ( Figure 2).

The study areas for this research project were selected based on specific criteria. Firstly, the researchers had prior experience of working on one of the stretches, which is the Pune-Sholapur Section of NH-9 in km.144/400 to Km. 249/000 in the State of Maharashtra. This experience could have provided insights and knowledge that could be useful in conducting the study.

Additionally, data was also provided by the same concessionaire as of the previous stretch on request for another stretch, which is the Six-Laning of Barwa-Adda-Panagarh Section of NH-2 from km 398.240 to km 521.120 including Panagarh Bypass in the States of and West Bengal. This data could have been relevant to the research objectives and could have assisted in achieving the desired outcomes.

## Methods

The proposed methodology for this research involves the following steps for implementing a random forest model machine learning technique for the accident severity prediction.

Data Preparation: The first step in implementing a random forest model for accident severity prediction is to collect and prepare data. Raw data of road accidents for the selected stretches of the highway can be obtained from secondary sources such as the Ministry of Road Transport and Highways (MoRTH) and National Highways Authority of India (NHAI). ^2^ Data wrangling and mining techniques can be used to clean and preprocess the data.

Feature Selection: Once the data is prepared, selecting appropriate features for the model becomes crucial. Feature selection plays a vital role in reducing the dimensionality of the data and enhancing the model’s accuracy. There are several techniques available for feature selection, such as statistical tests, correlation analysis, and principal component analysis (PCA). ^20^

Model Training: In the next step, a random forest model can be trained on the preprocessed data. The model can be developed using a machine learning based framework, as described in Breiman’s work on random forest. ^21^ The RF algorithm involves bagging and random feature selection techniques to create multiple decision trees that are aggregated to form a stronger learner. ^22^

Parameter Tuning: To improve the performance of a random forest model, it is important to fine-tune its parameters. The three key parameters that significantly impact the tuning performance of the random forest model are the total number of trees (n_estimators), the number of features used for each node segmentation (max_feature), and the maximum depth of a tree (max_depth). ^23^

Model Evaluation: After training the random forest model and optimizing its parameters, it is important to evaluate the model’s performance. Various evaluation metrics can be used, including accuracy, precision, recall, F1 score, and Area Under the Curve - Receiver Operating Characteristis (AUC-ROC) curve. ^24^

Model Implementation: Once the model has been trained and evaluated, it can be deployed for accident severity prediction. The methodology can be designed using python for building the model and forecasting the severity of road traffic accidents on Indian highways.

### Source data

Data on road accidents from selected stretches of highways was obtained from the Concessionaires of the National Highways Authority of India (NHAI) for two projects: Pune-Solapur and Bengal (BAEL) Section. For the Pune-Solapur Section of NH-9, which is located between km. 144/400 and km. 249/000 in the state of Maharashtra, accident dates from 2013 to 2018 were used. For the Six-Laning of Barwa-Adda-Panagarh Section of NH-2, which includes Panagarh Bypass and is located in the States of Jharkhand and West Bengal Stretch, accident dates from 2015 to 2019 were used for the stretch between km 398.240 and km 521.120. The raw data was subject to exploratory data analysis, as detailed in the following section.

### Data Preparation

In this stage, data gathering and exploration is performed using secondary source data. The dataset consists of 3257 observations out of which the 1855 observations are of Bengal (BAEL) Section and 1402 observations are of Pune- Solapur and 32 variables, including the target variable “accident severity.” The 32 attributes and their corresponding mappings are presented in Table 1.

**Table 1.** Dataset Attributes and Parameters Mapping.

Sl No	Attributes	Mapping
1	Accident Index	
2	Date	
3	Day of Week	1-Sunday, 2-Monday, 3-Tuesday, 4-Wednesday, 5- Thursday, 6-Friday, 7-Saturday
4	Time of Accident, Accident Location-A	1-Urban, 2-Rural, 3-Unallocated
5	Accident Location-A Chainage-km	
6	Accident Location-A Chainage-km-RoadSide	LHS, RHS
7-9	Nature of Accident-B1, Nature of Accident- B2, Nature of Accident-B3	1-Overturning, 2-Head on collision, 3-Rear End Collision, 4-Collision Brush/Side Wipe, 5-Right Turn Collision, 6- Skidding, 7a-Others-Hit Cyclist, 7b-Others-Hit Pedestrian, 7c-Others-Hit Parked Vehicle, 7d-Others-Hit Fixed Object, 7e-Others-Wrong Side Driving, 7f-Others-Hit Animal, 7g- Others-Hit Two Wheeler, 7h-Others-Unknown, 7i-Others- Fallen down, 8-Overtaking vehicle, 9-Left Turn Collision
10	Accident Severity -C	1-Fatal, 2-Grevious Injury, 3-Minor Injury, 4-No Injury
11-13	Classification of Accident-C1, Classification of Accident-C2, Classification of Accident-C3	1-Fatal, 2-Grevious Injury, 3-Minor Injury, 4-Non - Injury (Damage only)
14-18	Causes-D1, Causes-D2, Causes-D3, Causes- D4, Causes-D5	1-Drunken, 2-Overspeeding, 3-Vehicle out of control, 4a- Fault of driver of motor vehicle, 4b-Driver of other vehicle, 4c-Cyclist, 4d-Pedestrian, 4e-Passenger, 4f-Animal, 5a- Defect in mechanical condition of motor vehicle, 5b-Road condition
19	Road Feature-E	1-Single lane, 2-Two lanes, 3-Three lanes or more without central divider median, 4-Four lanes or more with central divider alongwith carriageway width
20	Road Condition-F	1-Straight Road, 2-Slight Curve, 3-Sharp Curve, 4-Flat Road, 5-Gentle incline, 6-Steep incline 7-Hump, 8-Dip
21	Intersection Type-G	1-T Junction, 2-’Y Junction, 3-’Four arm junction, 4- Staggered junction, 5-Roundabout, 6-Uncontrolled junction
22	Weather Conditions-H	1-Fine, 2-Mist/Fog 3-Cloud, 4-Light Rain, 5-Heavy Rain, 6-Hail/sleet, 7- Snow, 8-Strong Wind, 9-Dust Storm 10-Very Hot, 11-Very Cold, 12-Other extraordinary weather condition
23-26	Vehicle Type Involved-J-V1, Vehicle Type Involved-J-V2, Vehicle Type Involved-J-V3, Vehicle Type Involved-J-V4	1-Car/Jeep/Van, 2-SUV, 3-Bus, 4-Mini Bus, 5-Truck, 6- Two Wheeler, 7-Three Wheeler, 8-Cycle, 9-Pedestrian, 10- Tractor, 11-Unknown, 12-Animal, 13-Objects, 14-LCV, 15- MAV
27	Number of Vehicles	
28	Number of Casualties-Fatal	
29	Number of Casualties-Grievous Injury	
30	Number of Casualties-Minor Injury	
31	Number of Casualties-Non Injured	
32	Number of Casualties	

### Data Modelling

The random forest classification algorithm has been employed in this study to forecast the severity of road traffic accidents in India. This section details the procedure for implementing the model, performance evaluation, and discuss the results obtained. The random forest algorithm is written using python programming language.

The target variable for the random forest model is selected as the’Accident Severity’ which has classes as Fatal, Grevious Injury, Minor Injury and No Injury and indexed as [1-Fatal, 2-Grevious Injury, 3-Minor Injury, 4-No Injury.

The dataset is partitioned into training and testing sets with a ratio of 80% and 20%, respectively. The hyperparameters’n_estimators’ and’max_depth’ are specified, and a grid search is conducted with cross-validation (cv=5) to identify the optimal hyperparameters. The best parameters and scores are obtained. The best estimator is fit on the training data. Predictions are made on the test data and the accuracy of the model is obtained.

The algorithm and programme for Accident Severity Modelling using random forest are written in the Python programming language, and the code is made available to the public for further development. The source code can be accessed via the software availability statement.

Accuracy analysis on test data: Three metrics were employed to evaluate the effectiveness of the algorithms: accuracy, precision, and recall. These metrics are defined as follows:

Accuracy: The formula for a metric that measures the proportion of correctly predicted observations to the total number of observations is represented as:

$$\left(T\;P+T\;N\right)/\left(T\;P+T\;N+F\;P+\mathrm{FN}\right)$$

Precision is a metric that indicates the ratio of correctly predicted positive observations to the total number of predicted positive observations, and is calculated using the formula:

$$T\;P/\left(T\;P+F\;P\right)$$

Recall is a metric that reflects the ratio of correctly predicted positive observations to the total number of actual positive observations, and is determined using the formula:

$$T\;P/\left(T\;P+\mathrm{FN}\right)$$

## Result and Discussion

### Model Performance

The classification model used three hyperparameters -’max_depth’: 10,’max_features’:’sqrt’, and’n_estimators’: 100, and the results generated a confusion matrix for the training set. The matrix indicated the number of correctly and incorrectly classified instances for each class. The classification report provided precision, recall, and f1-score for each class, along with support. The model showed high precision and recall for class 1 but low precision and recall for classes 2, 3, and 4, with an overall accuracy of 67% and a weighted average f1-score of 0.64 on the training set. The macro average f1-score, which assigns equal weight to each class, was 0.53.

The optimal parameters for a random forest classifier model were determined through a grid search, with a max depth of 2, n estimators of 5000, and a random state of 0. The model was then applied to the test data, and the predictions were saved in an Excel file called “predicted output3.xls” for further analysis. The accuracy of the model on the test data was determined to be 0.4147, or approximately 41.47%, indicating that it accurately predicted the severity of traffic accidents in about 41.47% of test cases.

**Predicted outputs**

**Comparative analysis of observed and predicted accident severity index against dates**

The actual accident severity indices are represented by the observed values, while the predicted values are generated by the random forest model using the input features.

**Figure 3.**
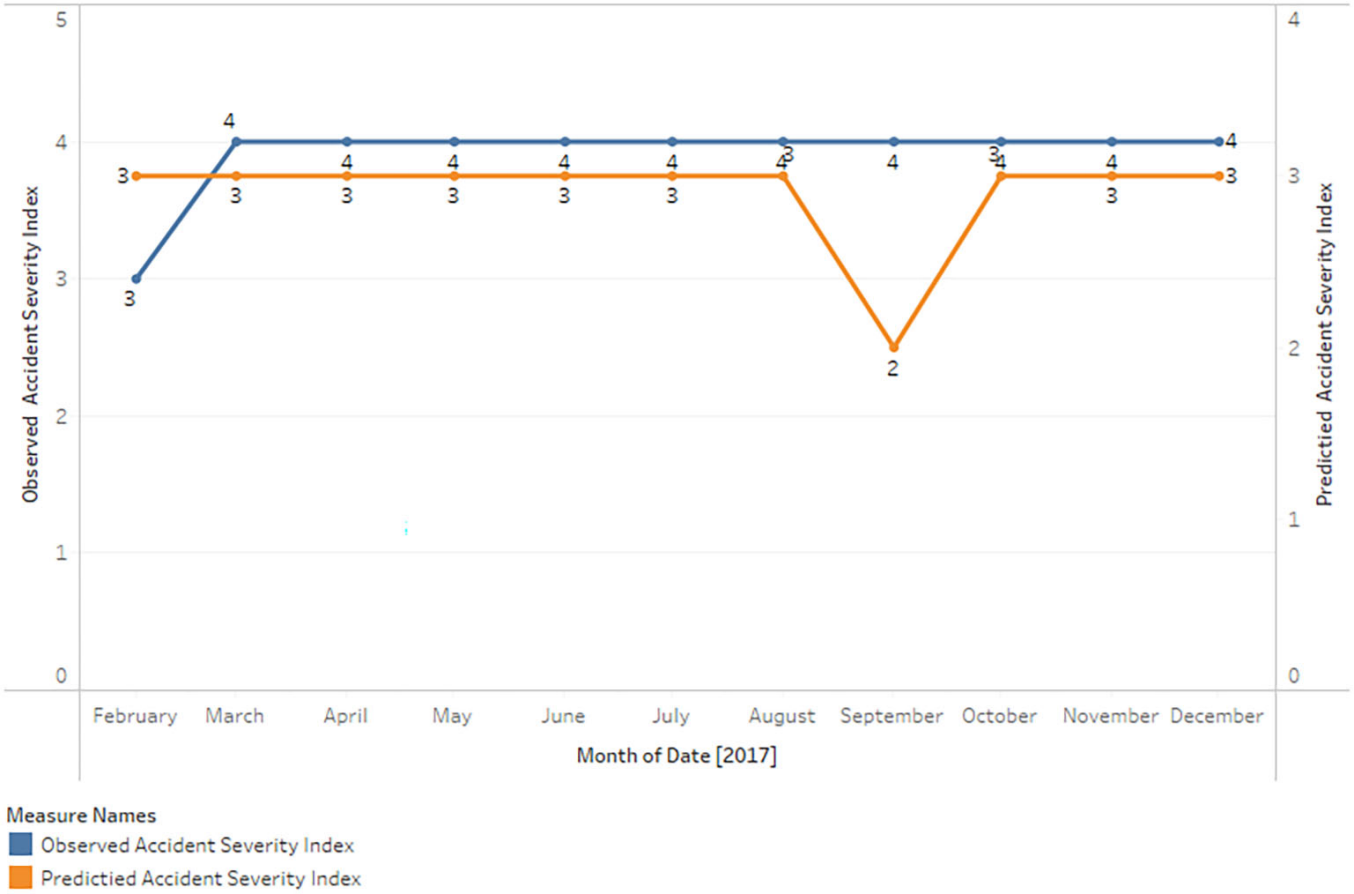
Comparison between Observed and Predicted Accident Severity Index.

The following is a summary ( Figure 3) of the comparison between the observed and predicted values:

On dates such as 25-02-2017, 17-04-2017, and 22-04-2017, the random forest model accurately predicts the accident severity index.

In a number of instances, the model predicts a lower accident severity index value than the observed value. 18-02-2017, 23-02-2017, and 27-03-2017, for example.

Occasionally, the model overestimates the accident severity index by predicting a higher value than the observed value, as on 24-05-2017 and 20-10-2017.

In general, the model frequently predicts a severity index of 2 for accidents, even when the observed values are distinct. This may indicate a bias in the model, possibly as a result of an imbalance in the training dataset, in which severity index 2 occurs more frequently than other categories.

**Comparative analysis of observed and predicted accident severity index against time**

**Figure 4.**
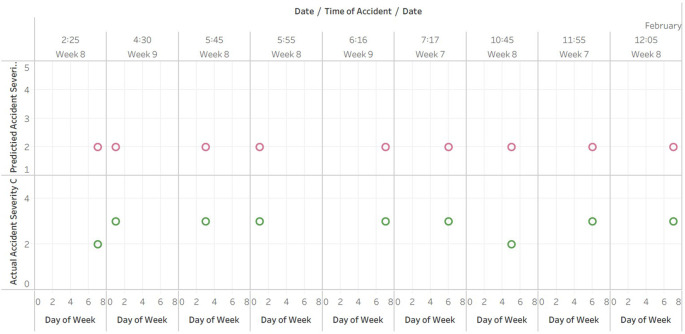
Comparative analysis of observed and predicted accident severity index against time.

Figure 4 displays the date, day of the week, and time of the accident, as well as the observed and predicted accident severity indices. The plotted for the 165 rows of predicted data doesn’t fit in A4 sheet hence the data is published and the link is provided in the Tableau graphs visuals availbility [i].

The dataset contains accident data from February 18, 2017 to December 31, 2017, as determined by Tableau analysis of the plot generated from the provided Excel table.

The observed accident severity index ranges from 1 to 4, where 1 corresponds to the least severe accident and 4 to the most severe accident.

The observed severity index for the vast majority of accidents in the dataset is 3, followed by 4. 2 indicates a less severe accident, while 4 indicates a more severe accident.

The majority of accidents within the dataset have a predicted severity index of 2, followed by an index of 1.

The analysis of the scatter plot reveals that the predicted severity index is typically lower than the observed severity index. This suggests that the model used to predict the severity of accidents is not always accurate and could be improved.

### Comparative analysis of observed and predicted accident severity index against Location and Chainages- RHS

**Figure 5.**
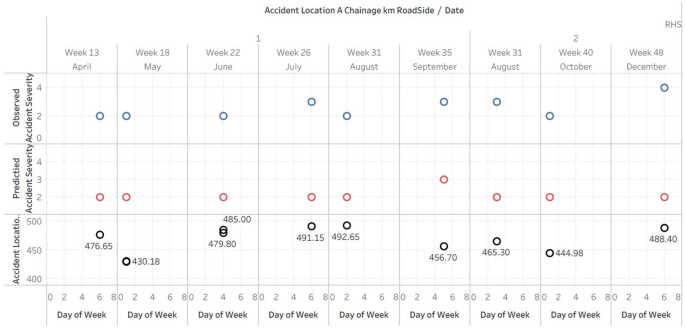
Comparative analysis of observed and predicted accident severity index against Location and Chainages-RHS.

The Tableau plot ( Figure 5) presents a detailed visual analysis of accident data on the right-hand side of the road. The data is organized by date and day of the week, displaying the accident location, observed accident severity index, and predicted accident severity index for each incident. The plot effectively illustrates the spatial distribution of accidents and their severity over time, enabling the identification of patterns and trends. The Tableau plot doesn’t fit in A4 sheet hence the data is published and the link is provided in the Tableau graphs visuals availability [ii].

It is evident from the analysis that the majority of accidents have an observed severity index of 2 or 3, indicating a moderate severity. However, the predicted accident severity index largely remains at 2, indicating that the predictions may be somewhat conservative and do not fully capture the observed severity range.

In addition, there appears to be no correlation between the day of the week and the frequency or severity of accidents across the different days of the week. This may suggest that external factors, such as traffic patterns or weather conditions, have a greater impact on the occurrence and severity of accidents than the day of the week.

### Comparative analysis of observed and predicted accident severity index against Location and Chainages- LHS

**Figure 6.**
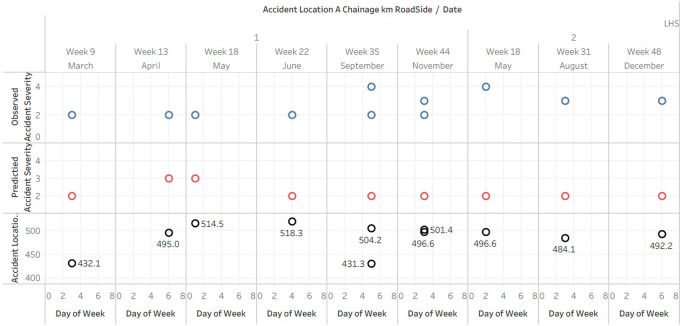
Comparative analysis of observed and predicted accident severity index against Location and Chainages-LHS.

The graph displays ( Figure 6) the date, day of the week, and accident location on Left Hand Side (LHS) of the road, as well as the observed and predicted accident severity indices. The plotted of predicted data doesn’t fit in A4 sheet hence the data is published and the link is provided in the Tableau graphs visuals availability [iii].

The scatterplot reveals that the majority of accidents on the left side of the road had a severity index of 2 or 3, with only a few instances of severity index 1 and 4. This indicates that the majority of collisions on the left side of the road were of moderate severity.

In the majority of cases, the predicted accident severity index was 2, with only a few instances of values 3 and 4. This suggests that the predictive model may be biased towards predicting less severe accidents.

There was no discernible pattern or trend between the day of the week and the occurrence of accidents. Accidents appeared to occur every day, indicating that the day of the week may not be a significant predictor of accident severity on the left side of the road.

The accident locations, as measured by Accident Location A Chainage km, were scattered along the roadway at various distances. This suggests that there may not be a particular accident hotspot or concentration on the left-hand side of the road.

### Data Recording and availability

The recording of road accident data in India must comply with the MoRTH & IRC guidelines, utilizing the Road Accident Recording and Reporting Formats. Despite this, there exists a need for a more advanced data recording system to effectively model road safety. The digital monitoring of road accidents can increase the frequency of data collection and minimize the absence of crucial information. Often, the lack of a system or individual to document the accident leads to the absence of important road accident data. This missing data can be regained through the use of machine learning, thus enhancing the accuracy of road safety modeling.

## Conclusion

The random forest classifier model predicted the severity of traffic accidents with an overall accuracy of 67% on the training set and approximately 41.47% on the test set. Indicating possible bias or imbalance in the training dataset, the model tended to predict a lower severity index than the observed values. There were no discernible relationships between the day of the week and the occurrence or severity of accidents. The performance of the model can be enhanced by correcting the dataset imbalance and refining the model’s hyperparameters.

The observed and predicted accident severity indices were compared against a number of variables, including dates, times, and locations on both sides of the road. In some instances, the model accurately predicted the accident severity index, but it frequently underestimated accident severity. No discernible patterns or trends were observed in terms of accident location, indicating that external factors may have a greater influence on the occurrence and severity of accidents.

To improve road safety modelling, it is essential to adopt a more sophisticated data recording system consistent with MoRTH and IRC recommendations. Digital monitoring of road accidents can increase the frequency of data collection and reduce the loss of vital information. Integrating machine learning techniques can contribute to more effective interventions and decision-making in the field of traffic accident prevention and mitigation.

## Future Scope

The study presented provides a good starting point for future research in the field of road safety modeling and accident prevention for Indian highways. However, with the limitations of the present study there opens potential areas for future research as mentioned below which will be taken up in continuation.

Dataset improvement: The study identified the possibility of dataset bias and imbalance affecting model performance. Future research will focus on improving the quality and quantity of data, reducing bias and improving model performance. This will involve exploring alternative data sources, enhancing data collection methods, and addressing data quality issues.

Model improvement: The study used the random forest algorithm to develop a predictive model for traffic accident severity. In future research, other machine learning algorithms or ensemble models to improve model performance will be explored. Additionally, refining hyperparameters and addressing dataset imbalance will be done to improve model accuracy.

External factors analysis: The study highlighted the influence of external factors on accident severity prediction. Future research can focus on exploring the impact of external factors such as weather conditions, road infrastructure, and driver behavior on accident severity. This can enhance the accuracy of predictive models and inform decision-making in accident prevention efforts.

Real-time monitoring: The study highlighted the need for a sophisticated data recording system in line with MoRTH and IRC guidelines. Future research can focus on developing a real-time monitoring system that can capture road safety data in real-time and provide insights for accident prevention efforts.

## Data Availability

Zenodo. Data for Accident Severity Prediction Modelling for Indian Highways Case Study, https://doi.org/10.5281/zenodo.7773156. ^25^ This project contains the following underlying data:
•
Accdataset_hk_PS_BAEL_Combined.csv (The dataset consists of 3257 observations out of which the 1855 observations are of Bengal (BAEL) Section and 1402 observations are of Pune- Solapur.) •
predicted_output_1.xlsx (This is level-2 processed data derived from raw accident data using prediction modeling. The data has been indexed from 1 to 4 for further analysis, and there are a total of 165 rows in the predicted output observations. Data are available under the terms of the Creative Commons Attribution 4.0 International license (CC-BY 4.0).

## Software availability

• Github: https://github.com/humera-k/RF_Accident_Severity
• https://zenodo.org/badge/latestdoi/616376786

Data are available under the terms of the Creative Commons Attribution 4.0 International license (CC-BY 4.0).

## Tableau graphs visuals availability

1. Accidental_Analysis_1 | Tableau Public (Comparative analysis of observed and predicted accident severity index against time)
2. Accidental_Analysis_1 (Comparative analysis of observed and predicted accident severity index against Location and Chainages-Right hand Side (RHS))
3. Accidental_Analysis_1 (Comparative analysis of observed and predicted accident severity index against Location and Chainages-Left Hand Side (LHS))
