# Supplementary material for: Accident severity prediction modeling for road safety using random forest algorithm: an analysis of Indian highways
Source: F1000Res. 2023 Oct 20;12:494. Originally published 2023 May 15. [Version 2] doi: 10.12688/f1000research.133594.2 (PMC10787871; doi:10.12688/f1000research.133594.2)
Supplement: Peer review history

# Reviewer report: Reviewer response for version 2

**DOI:** 10.5256/f1000research.157426.r217365

**Published:** 2024-01-22

**Authors:** Sanjeev Sinha

**Affiliations:** 1 Department of Civil Engineering, National Institute of Technology Patna, Patna, Bihar, India

The authors have responded to all the comments satisfactorily. I do not have any further comments.

Is the work clearly and accurately presented and does it cite the current literature?

Partly

If applicable, is the statistical analysis and its interpretation appropriate?

Partly

Are all the source data underlying the results available to ensure full reproducibility?

Partly

Is the study design appropriate and is the work technically sound?

Yes

Are the conclusions drawn adequately supported by the results?

Partly

Are sufficient details of methods and analysis provided to allow replication by others?

Partly

Reviewer Expertise:

Traffic Engineering, Transportation Planning, Highway Materials, Logistics

I confirm that I have read this submission and believe that I have an appropriate level of expertise to confirm that it is of an acceptable scientific standard.

# Reviewer report: Reviewer response for version 2

**DOI:** 10.5256/f1000research.157426.r228819

**Published:** 2024-01-13

**Authors:** Holman Ospina-Mateus

**Affiliations:** 1 Universidad Tecnologica de Bolivar, Cartagena, Bolívar, Colombia

The article proposes an analysis of road safety in the context of India, using a random forest methodology, as a proposal to predict conditions on highways. Below are some aspects that should be improved within the article:

 • In the abstract, it is noted: “AI models”, random forest is a traditional machine learning method.

 • The results in the abstract: They can be written better.

 • Road accident datasets are traditionally unbalanced and may be subject to considering sampling techniques that allow them to be balanced or leveled.

 • Before explaining the method, it would be good to see an overview in descriptive statistics of the country's official data on road safety, where the participation by road actors, spatiotemporal conditions, and sociodemographic conditions can be seen.

 • Figure four has errors in structure, labels, and legends.

 • In the analysis and relationships that the variables may have, there is no significant contribution.

 • It is recommended within the model to reduce the categories of the variables, seeking better relational results.

 • The ROC curve is not observed.

 • No technique was considered to balance accident sampling in relation to predictions.

 • The model suffers from a high hyper parameterization; the authors did not validate a pre-analysis that linked or filtered the variables that could be part of the model.

 There was a lack of clear discussion at the end of the document, contrasting the results with similar investigations.

Is the work clearly and accurately presented and does it cite the current literature?

Yes

If applicable, is the statistical analysis and its interpretation appropriate?

Partly

Are all the source data underlying the results available to ensure full reproducibility?

Yes

Is the study design appropriate and is the work technically sound?

Yes

Are the conclusions drawn adequately supported by the results?

Yes

Are sufficient details of methods and analysis provided to allow replication by others?

Yes

Reviewer Expertise:

Road safety.

I confirm that I have read this submission and believe that I have an appropriate level of expertise to confirm that it is of an acceptable scientific standard, however I have significant reservations, as outlined above.

# Reviewer report: Reviewer response for version 2

**DOI:** 10.5256/f1000research.157426.r217364

**Published:** 2023-11-01

**Authors:** Hamsa Zubaidi

**Affiliations:** 1 Roads and Transport Engineering Department, University of Al-Qadisiyah, Diwaniyah, Al-Qādisiyyah Governorate, Iraq

The authors addressed my comments, thank you.

Is the work clearly and accurately presented and does it cite the current literature?

Yes

If applicable, is the statistical analysis and its interpretation appropriate?

Yes

Are all the source data underlying the results available to ensure full reproducibility?

Partly

Is the study design appropriate and is the work technically sound?

Yes

Are the conclusions drawn adequately supported by the results?

Partly

Are sufficient details of methods and analysis provided to allow replication by others?

Partly

Reviewer Expertise:

Transportation Safety

I confirm that I have read this submission and believe that I have an appropriate level of expertise to confirm that it is of an acceptable scientific standard.

# Reviewer report: Reviewer response for version 1

**DOI:** 10.5256/f1000research.146596.r173654

**Published:** 2023-10-05

**Authors:** Sanjeev Sinha

**Affiliations:** 1 Department of Civil Engineering, National Institute of Technology Patna, Patna, Bihar, India

The work is clearly and accurately presented however, the motivation behind the work and its academic contribution needs to be highlighted.  

 Though relevant literature has been cited, it has not been described for its application in the present study. Further, there is some literature which seems not to be correctly referred and cited. As example the references, 7, 14 and 15 needs to be re-checked.   

 The title of the work is development of prediction modeling for accident severity, the choice of factors, their contribution and transferability of the model developed needs to be highlighted.

 The literature review shows several works related to the topic thus novelty of the work also need to be highlighted.

Is the work clearly and accurately presented and does it cite the current literature?

Partly

If applicable, is the statistical analysis and its interpretation appropriate?

Partly

Are all the source data underlying the results available to ensure full reproducibility?

Partly

Is the study design appropriate and is the work technically sound?

Yes

Are the conclusions drawn adequately supported by the results?

Partly

Are sufficient details of methods and analysis provided to allow replication by others?

Partly

Reviewer Expertise:

Traffic Engineering, Transportation Planning, Highway Materials, Logistics

I confirm that I have read this submission and believe that I have an appropriate level of expertise to confirm that it is of an acceptable scientific standard, however I have significant reservations, as outlined above.

# Response

**Published:** 2023-10-12

**Authors:** Humera Khanum

**Comment 1: **The work is clearly and accurately presented however, the motivation behind the work and its academic contribution needs to be highlighted.

**Author's Response: **Thank you for your constructive feedback. We agree that highlighting the motivation and academic contribution of this work is crucial for a well-rounded understanding of the study. In the revised manuscript, we have included a section in the Introduction where we elaborate on the motivation behind this work, particularly focusing on the rising number of road accidents and the necessity to develop predictive models to understand and mitigate accident severity. Additionally, we have outlined the academic contribution of our study in the Discussion section, emphasizing the novelty and practical implications of our findings.

**Comment 2: **Though relevant literature has been cited, it has not been described for its application in the present study. Further, there is some literature which seems not to be correctly referred and cited. As example the references, 7, 14 and 15 needs to be re-checked.  

**Author's Response: **We appreciate your attention to detail. We have revisited the cited literature, especially references 7, 14, and 15, and corrected the citations as suggested. Moreover, we have expanded the section to better explain the relevance and application of the cited works in the context of our study.

**Comment 3: **The title of the work is Development of prediction modeling for accident severity, the choice of factors, their contribution and transferability of the model developed needs to be highlighted.

**Author's Response: **Thank you for your suggestion. In the revised manuscript, we have dedicated a subsection within the Methodology section to discuss the choice of factors, their contribution, and the transferability of the developed model.  

**Comment 4:**The literature review shows several works related to the topic thus novelty of the work also need to be highlighted.

**Author's Response: **We value your input on highlighting the novelty of our work. In the revised manuscript, we have included a paragraph at the end in the conclusion section where we delineate the novelty of our study in comparison to existing literature. We have emphasized the innovative aspects of our predictive model and how our work contributes uniquely to the understanding and prediction of accident severity.

# Reviewer report: Reviewer response for version 1

**DOI:** 10.5256/f1000research.146596.r202786

**Published:** 2023-09-18

**Authors:** Hamsa Zubaidi

**Affiliations:** 1 Roads and Transport Engineering Department, University of Al-Qadisiyah, Diwaniyah, Al-Qādisiyyah Governorate, Iraq

1. The author mentioned "accident" in the paper,  "Accident" is a broader term that implies an unintentional or unforeseen incident. It can encompass various events, including crashes, collisions, or other incidents resulting from human error, mechanical failure, or environmental factors. "Crash" is a more specific term that typically refers to a collision or impact between vehicles, objects, or individuals. It often implies a sudden and forceful event.
  Ultimately, the choice between "accident" and "crash" should be based on the specific details and nature of the event being described in the paper, and relying on that "accident" should be replaced by "crash".
2. The author should add a random forest formula in the methodology.
3. It might be better if there is a Gini impurity test in the paper to understand the importance of explanatory variables.

Is the work clearly and accurately presented and does it cite the current literature?

Yes

If applicable, is the statistical analysis and its interpretation appropriate?

Yes

Are all the source data underlying the results available to ensure full reproducibility?

Partly

Is the study design appropriate and is the work technically sound?

Yes

Are the conclusions drawn adequately supported by the results?

Partly

Are sufficient details of methods and analysis provided to allow replication by others?

Partly

Reviewer Expertise:

Tansportation Safety

I confirm that I have read this submission and believe that I have an appropriate level of expertise to confirm that it is of an acceptable scientific standard, however I have significant reservations, as outlined above.

# Response

**Published:** 2023-09-29

**Authors:** Humera Khanum

**Comment:1 **The author mentioned "accident" in the paper, “Accident" is a broader term that implies an unintentional or unforeseen incident. It can encompass various events, including crashes, collisions, or other incidents resulting from human error, mechanical failure, or environmental factors. "Crash" is a more specific term that typically refers to a collision or impact between vehicles, objects, or individuals. It often implies a sudden and forceful event.

 Ultimately, the choice between "accident" and "crash" should be based on the specific details and nature of the event being described in the paper, and relying on that "accident" should be replaced by "crash".

**RESPONSE: **Thank you for your insightful comments and for bringing up the concern regarding the usage of the term "accident" in our paper. Your observation about the broad implication of "accident" and the specificity of "crash" is indeed valid.

 I would like to clarify that in the context of our paper, the mention of "accidents" is based on Indian data, which is commonly referred to as road traffic accident data in official and legal documents in India. The term "accident" here encompasses various events, including crashes, collisions, or other incidents resulting from human error, mechanical failure, or environmental factors, as per the definition used in the Indian context.

 All the parameters mentioned in the paper have been taken into consideration, and the terminology used is consistent with the data source and regional context. We appreciate the suggestion to use the term "crash" for more specificity and will certainly consider this in our future research and writings, ensuring that the terminology aligns with the global standards and is clear to all readers.

**Comment 2:** The author should add a random forest formula in the methodology.

**RESPONSE: **The random forest formula is added to the methods section as below:

 Random Forest Algorithm Formulation: The Random Forest algorithm can be represented as:

 [RF(X) = (1/B) * Σ(T_b(X)) from b=1 to B]

 where X are the input features, B is the number of trees, and T_b(X) is the prediction of the b-th individual decision tree.

**Comment 3:** It might be better if there is a Gini impurity test in the paper to understand the importance of explanatory variables.

**RESPONSE: **The random forest formula is added to the methods section as below:

  In the construction of the Random Forest model for predicting accident severity, Gini impurity is employed as a criterion to evaluate the significance of different explanatory variables. Gini impurity, a measure utilized within the framework of decision trees (the base learners in a Random Forest), is crucial for the optimal selection of features at each node split. It offers a quantitative metric to discern the effectiveness of a variable in segregating the target classes.

 Mechanism of Gini Impurity: In the context of binary classification, the Gini impurity for a node is calculated as:

*I* _G_ *(p)=* *k=1* *n* *p* *2*k 

 where, *pk*​ is the proportion of samples classified to class *k* at that node, and the summation operates over all classes. A lower Gini impurity score suggests a higher purity of the node, indicating an enhanced classification.

 Gini Importance in Random Forest: In the developed Random Forest model, the Gini impurity plays a dual role: **Node Splitting**: It aids in the identification of the most significant variable at each node by evaluating the potential reduction in impurity for each split, and **Feature Importance**: Post model training, the average decrease in impurity caused by each feature across all trees is computed, known as Gini importance. This metric offers insights into the relative significance of different features for the prediction task.
